# Development of a Low-Cost Portable Exhaled Breath Ammonia Detector for Supplementary Five-Stage CKD Classification Using Embedded Threshold Logic

**DOI:** 10.3390/s26175371

**Published:** 2026-08-25

**Authors:** Winda Astuti, Juan Alexander Kwan, Elioenai Sitepu, Syauqi Abdurrahman Abrori, Feri Setiawan

**Affiliations:** 1 Automotive and Robotics Program, Computer Engineering Department, BINUS ASO School of Engineering, Bina Nusantara University, Jakarta 11480, Indonesia; juan.kwan@binus.ac.id (J.A.K.); syauqi.abrori@binus.ac.id (S.A.A.); 2 Industrial Engineering Department, Graduate Program, Master of Industrial Engineering, Bina Nusantara University, Jakarta 11480, Indonesia; elioenai.sitepu@binus.ac.id; 3 Computer Science Department, Bina Nusantara University, Jakarta 11480, Indonesia; feri.setiawan@binus.edu

**Keywords:** breath ammonia, chronic kidney disease, CKD staging, non-invasive diagnosis, embedded system, MQ-137, sex-specific algorithm, MDRD, machine learning, point-of-care, eGFR

## Abstract

**Highlights:**

**What are the main findings?**

- A sex-adjusted breath ammonia staging algorithm—applying distinct MDRD-based thresholds for male and female patients—was developed using two independent clinical datasets, achieving dataset-level classification accuracy of 82% (male) and 92% (female).
- A low-cost, portable embedded prototype using a commercially available ammonia gas sensor and Arduino Nano achieved hospital testing accuracy of 90.5% for male patients and 71.4% (5/7) for female patients, demonstrating real-world screening feasibility.

**What are the implications of the main findings?**

- Breath ammonia-based CKD staging with sex-adjusted MDRD thresholds offers a non-invasive, accessible complement to blood-based diagnostics, particularly suited for resource-limited settings.
- The discrepancy between dataset-level and clinical accuracy highlights the need for larger, balanced clinical cohorts in future validation studies.

**Abstract:**

Conventional diagnosis of chronic kidney disease (CKD) relies predominantly on invasive blood-based examinations, limiting the scalability of kidney health screening in resource-constrained environments. This study presents embedded engineering framework for non-invasive, breath-based CKD staging framework supported by machine learning and implemented on a low-cost embedded platform. To account for physiological sex differences in baseline creatinine production, estimated glomerular filtration rate (eGFR) values and breath ammonia concentrations were derived from two independent clinical cohorts using sex-specific MDRD equations (incorporating the standard male formula and the 0.742 female correction factor, respectively) and creatinine–BUN conversion models, with male- and female-parameterized algorithms developed in parallel. The resulting feature space was analyzed using four unsupervised clustering approaches to stratify subjects into five clinically meaningful kidney function stages. Stage-specific ammonia thresholds were implemented within an Arduino Nano-based prototype equipped with an MQ-137 gas sensor and OLED display, enabling real-time point-of-care classification. Dataset-level classification accuracy reached 82% for the male algorithm and 92% for the female algorithm. Hospital-based validation on 29 patients (22 male, 7 female) yielded a real-world testing accuracy of 90.5% (20/22) for male patients and 71.4% (5/7) for female patients, a discrepancy largely attributable to the small female sample size. Because the current evaluation lacks healthy control subjects and is constrained by sample size, these empirical results serve primarily to demonstrate hardware-software functional integration and real-world deployment feasibility rather than definitive clinical efficacy. Despite these preliminary, sample-limited clinical datasets, results suggest this approach holds promise as an accessible, non-invasive screening complement to conventional diagnostic pathways, particularly in low-resource healthcare settings.

## 1. Introduction

Chronic kidney disease (CKD) has emerged as one of the most pressing non-communicable disease burdens of the 21st century. Global estimates from the 2023 Global Burden of Disease Study indicate that approximately 788 million adults worldwide were living with CKD in 2023—more than double the 378 million recorded in 1990—with an age-standardized prevalence of 14.2% across 204 countries and territories [1]. This trajectory places CKD among the leading causes of mortality and disability-adjusted life-years globally, with a disproportionate and growing impact on low- and middle-income countries where access to nephrology care and laboratory infrastructure remains severely limited [2]. The burden is particularly acute in Eastern Europe and Central Asia, where CKD remains a leading contributor to premature mortality and years lived with disability [3].

Early detection of CKD is clinically critical. Disease progression through Stages 1–5, defined by declining estimated glomerular filtration rate (eGFR), is largely silent in its initial phases yet amenable to intervention if identified promptly [4]. Conventional diagnostic pathways rely on serum creatinine and blood urea nitrogen (BUN) measurements derived from venous blood sampling, followed by eGFR calculation using validated equations such as the Modification of Diet in Renal Disease (MDRD) formula [5]. Kidney function is routinely assessed through a panel of blood-based markers including serum creatinine, BUN, and derived eGFR values [6], and advances in CKD diagnosis continue to be reviewed extensively in the literature [7]. While clinically robust, this approach carries inherent barriers to scalability. Laboratory processing introduces time delays, and the procedure itself is invasive—a non-trivial obstacle given the documented prevalence of needle phobia. An international survey of 2098 adults found that 63.2% reported experiencing needle phobia, with 52.2% of those affected actively avoiding blood draws consequently [8]. A systematic review and meta-analysis further confirmed that needle fear is prevalent across demographic groups and consistently associated with avoidance of venipuncture and other invasive procedures [9]. In populations requiring regular kidney function monitoring, such avoidance behavior can translate directly into delayed diagnosis and accelerated disease progression.

These limitations have motivated growing interest in non-invasive, breath-based diagnostic modalities. In CKD patients, impaired renal clearance leads to systemic accumulation of urea, which is subsequently hydrolyzed by salivary urease into ammonia (NH_3_), a volatile compound measurable in exhaled breath [10]. Among the numerous breath-borne volatile organic compounds investigated as CKD biomarkers, ammonia has emerged as the most clinically promising candidate. A range of sensing technologies have been evaluated for ammonia detection at clinically relevant concentrations, including plasmonic and electrochemical biosensors [11]. In a cohort study of 121 CKD patients spanning all five disease stages, Chan et al. demonstrated that breath ammonia concentration was significantly and progressively elevated across CKD stages (Stage 1–5: 636 ± 94 to 12,781 ± 1807 ppb), with an area under the ROC curve of 0.835 for distinguishing early-stage CKD and strong correlations with BUN, serum creatinine, and eGFR [12]. This finding was further corroborated by Romani et al., who reported that breath ammonia was the most accurate volatile organic compound for differentiating CKD patients from controls, achieving an Area Under the Curve (AUC) of 0.902 in a study of over 120 participants [13].

Building on this biochemical foundation, several research groups have developed breath ammonia sensing systems for increasing analytical sophistication [14,15,16,17]. Machine learning approaches have also been applied directly to clinical kidney disease datasets, with deep learning architectures demonstrating strong classification performance [18]. Optical approaches—including ultraviolet differential optical absorption spectroscopy combined with neural networks—have demonstrated high-precision detection of exhaled ammonia at parts-per-billion levels [17]. A flexible, room-temperature ammonia sensor with a detection limit of 100 ppb, evaluated against 96 clinical breath samples, achieved diagnostic accuracies of 0.93 and 0.94 for distinguishing CKD from healthy controls and staging disease severity respectively, using a support vector machine classifier [19]. Wearable approaches have also been explored, including arrays of resistive gas sensors embedded within disposable tight-fitting face piece (FFP2) facemasks, capable of simultaneously detecting ammonia and other CKD-related volatile metabolites [20]. A systematic review of electronic nose systems for kidney disease diagnosis further confirmed that breath-based approaches offer clinically meaningful and minimally invasive pathways for CKD characterization [21]. To contextualize the position of our proposed embedded device within the broader diagnostic landscape, Table 1 provides a comparative summary of reported breath ammonia and VOC sensing approaches for CKD evaluation. While advanced platforms—such as mass spectrometry (SIFT-MS), optical UV-DOAS, and custom flexible or polymer sensor arrays—achieve sub-ppb analytical precision, they rely on complex instrumentation or specialized fabrication that limits deployment in low-resource clinical settings. The trade-off in the present work is intentional: by combining off-the-shelf hardware (<USD 100) with sex-adjusted MDRD staging algorithms, our platform provides a portable, accessible preliminary screening tool for point-of-care triage where laboratory infrastructure is unavailable.

Despite their analytical precision, these systems share a common limitation: they rely on laboratory-grade or custom-fabricated sensing elements that are technically complex and costly to operate, making them unsuitable for deployment in resource-constrained clinical environments. Point-of-care testing (POCT) has demonstrated value in improving CKD detection rates in underserved communities, yet the approaches to kidney function assessment remain blood-based [22], perpetuating the invasiveness barrier. A low-cost, breath-based CKD staging device built from commercially available components—deployable without laboratory infrastructure—represents an important and largely unmet need, particularly in Southeast Asian and other lower-middle-income healthcare settings. Embedded sensor systems integrated with AI-driven decision pipelines have shown a clinical improvement with less cost in resource-constrained settings across Southeast Asia [23].

A further gap in the existing literature concerns sex differences in CKD algorithm design. While the epidemiology of CKD is well established to differ between sexes—with CKD prevalence higher in women but kidney failure progression faster in men [24]—the downstream implications for breath ammonia-based staging remain underexplored. These disparities extend to clinical management, where women with CKD are less likely to be referred to nephrology services or to receive appropriate monitoring despite higher disease prevalence [25]. Physiological differences between male and female patients, most notably the 0.742 correction factor applied to female patients in the MDRD eGFR equation to account for lower average muscle mass [5], directly affect the relationship between circulating creatinine, BUN, and measured breath ammonia. Despite this well-established physiological basis, none of the embedded or portable breath ammonia sensing systems reported to date have incorporated sex-differentiated diagnostic thresholds [14,15,16,17,18,19,20,21,22,23], and prior efforts toward embedded breath ammonia classification have so far been limited to male-only populations [26,27], leaving female-specific staging largely unaddressed in hardware-integrated systems.

This study addresses both gaps. A low-cost, portable, embedded breath ammonia sensing system incorporating sex-adjusted, MDRD-based CKD staging thresholds, derived via unsupervised machine learning applied in parallel to two independent clinical cohorts was developed. The system employs a commercially available MQ-137 ammonia gas sensor interfaced with an Arduino Nano microcontroller and an OLED display, enabling real-time five-stage CKD classification at the point of care. The principal contributions of this work are: (1) the derivation and validation of sex-adjusted ammonia thresholds—based on the male and female correction variants of the MDRD equation—applied in parallel to two independent clinical cohorts (Indian and Bangladeshi hospital datasets, respectively); (2) a systematic comparison of four unsupervised clustering approaches—K-Means, Agglomerative Hierarchical Clustering, DBSCAN, and Gaussian Mixture Models—for ammonia threshold determination; and (3) preliminary hospital-based validation on 29 real patients demonstrating the feasibility of the integrated hardware prototype as a practical screening complement to conventional blood-based diagnostics.

## 2. Materials and Methods

### 2.1. System Overview

**Figure 1:**
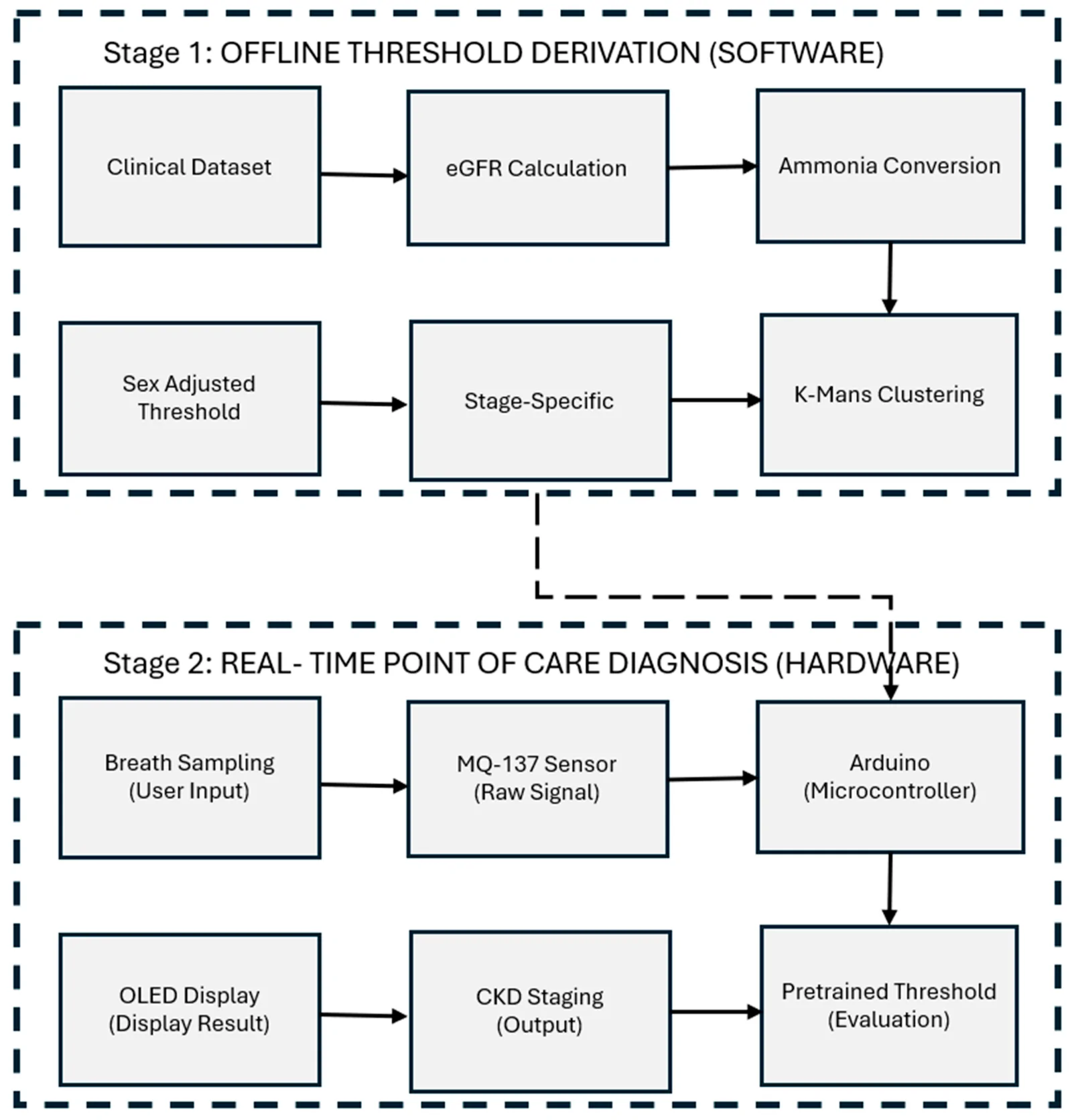
Overview of system architecture distinguishing between (1) offline software derivation of static sex-adjusted thresholds, and (2) real-time hardware diagnostic workflow starting from breath sampling to point-of-care OLED classification output.

The proposed system follows two distinct phases: an offline software derivation stage and a real-time online hardware execution stage, as shown in Figure 1. During the software stage, sex-adjusted staging thresholds are pretrained offline using machine learning clustering across two clinical datasets. Once derived, these static threshold values are embedded directly into the microcontroller firmware. During real-time operation, the hardware workflow begins directly with patient breath sampling, where exhaled ammonia concentrations are measured and immediately evaluated against the pretrained embedded thresholds to deliver real-time CKD staging.

### 2.2. Dataset and Preprocessing

This study utilizes two independent, publicly available clinical datasets of kidney disease patients: a cohort from an Indian hospital (UCI C5G020) and a cohort from several hospitals in Bangladesh (UCI C5WP64). Neither dataset contains individual-level sex labels; both are processed at the cohort level. The standard MDRD equation was applied to the Indian cohort, and the female-correction MDRD variant (×0.742) was applied to the Bangladeshi cohort, consistent with the convention used in prior work on this dataset pair [28,29]. The physiological justification for multiplying serum creatinine by the 0.742 coefficient stems from the seminal study by Levey et al. (1999) [5], which established the Modification of Diet in Renal Disease (MDRD) equation. Using multiple linear regression analysis across a large patient cohort, the researchers demonstrated that females produce significantly less endogenous creatinine than males at equivalent levels of renal function, primarily due to lower average muscle mass. Omitting this 0.742 multiplier would systematically overestimate the actual estimated glomerular filtration rate (eGFR) in female patients, making this female-specific correction essential for accurately diagnosing and staging chronic kidney disease [5]. Therefore, we refer to the resulting two algorithm variants as ‘male’ and ‘female’ to reflect the MDRD correction term applied consistent with standard clinical nomenclature for this formula rather than a verified sex distribution within either source cohort. Because the two cohorts also differ in geographic origin and population characteristics, threshold differences between the two algorithm variants should be interpreted with this caveat in mind; this is revisited in the Discussion (Section 4). The datasets were processed using Python(v3) on the Google Colab platform. Relevant variables—age, blood urea (ureum), serum creatinine, and classification labels—were extracted, and entries classified as non-CKD were removed. In one dataset, symbolic variable values were replaced with their corresponding constants, and range-expressed values were converted to their midpoint averages.

The primary objective of this embedded framework is to perform five-stage CKD risk stratification. Relevant variables—age, blood urea (ureum), serum creatinine, and classification labels—were extracted. Entries classified as non-CKD were excluded during model training to focus the unsupervised clustering specifically on establishing boundaries across established CKD severity stages. Consequently, the lowest concentration interval (Stage 1) corresponds to patients with eGFR $\geq 90{\;\mathrm{mL}/\min/1.73\;m}^{2}$ within a kidney disease cohort. The device is thus designed as a secondary staging tool for individuals identified as at-risk or undergoing nephrology evaluation, rather than a primary binary classifier for distinguishing healthy individuals from early-stage patients.

### 2.3. eGFR Calculation

Kidney function staging was performed using the estimated Glomerular Filtration Rate (eGFR), calculated via the Modification of Diet in Renal Disease (MDRD) equation [5]:

(1) $$eGFR\;=186\times \;{Serum\;creatinine\;(\frac{mg}{dL})}^{-1.154}\times {Age}^{-0.203}\times 0.742\;\left(if\;female\right)\times 1.212\;\left(if\;black\right),$$

Patients were categorized into five clinical CKD stages according to standard KDIGO criteria: Stage 1 (≥90), Stage 2 (60–89), Stage 3 (30–59), Stage 4 (15–29), and Stage 5 (<15 mL/min/1.73 m^2^) [4].

Several equations have been proposed for eGFR estimation; the MDRD equation was selected here for its sex-specific applicability and established use in the source literature [30,31]. The key distinction between male and female calculations lies in the application of a 0.742 correction factor to female patients. eGFR values were calculated by substituting each patient’s age and serum creatinine into the equation, with the female correction factor applied where applicable. It is noted that while the race-free CKD-EPI 2021 equation is now the recommended standard in clinical practice [32], the MDRD equation was retained in this study for consistency with the source datasets and the ammonia conversion formulas derived from Chan et al. [12], which were similarly based on MDRD-derived eGFR values. The five CKD stages were defined according to standard clinical criteria: Stage 1 (eGFR ≥ 90), Stage 2 (60–89), Stage 3 (30–59), Stage 4 (15–29), and Stage 5 (<15 mL/min/1.73 m^2^) [4].

### 2.4. Urea to BUN Conversion

The clinical datasets contain urea concentration values rather than blood urea nitrogen (BUN). Since the ammonia conversion formulas are expressed in terms of BUN, a molecular weight-based conversion was applied. Urea (CO(NH_2_)_2_) has a molecular weight of 60 g/mol and contains two nitrogen atoms (molecular weight 14 g/mol each), yielding the following relationship:

(2) $$BUN=\frac{Urea}{2.14}$$

### 2.5. Ammonia Conversion Formula

To estimate breath ammonia concentration for each patient, conversion formulas relating to serum creatinine and BUN to breath ammonia were derived from the correlation graphs reported by Chan et al. [12]. Because public datasets lacked direct breath measurements, estimated ${\mathrm{NH}}_{3}$ concentrations were derived from serum markers solely for initial threshold parameterization, introducing an inherent mathematical dependence. True sensor performance was evaluated exclusively via measured breath ${\mathrm{NH}}_{3}$ in an independent hospital testing cohort. Linear regression was applied to both the creatinine–ammonia and BUN–ammonia relationships, yielding:

(3) $$Ammonia\;\left(ppb\right)=\;1896.1\;\times \;Creatinine(mg/dL)\;-\;568.8$$

(4) $$Ammonia\;\left(ppb\right)=148.8\times BUN\;(mg/dL)-1488$$

The final ammonia value for each data entry was calculated by averaging the results of both equations.

### 2.6. Unsupervised Clustering

Four unsupervised clustering algorithms were evaluated to identify the optimal method for grouping patients into five CKD stages based on their eGFR values: K-Means Clustering, Agglomerative Hierarchical Clustering, DBSCAN, and Gaussian Mixture Models (GMM). K-Means and Agglomerative Hierarchical Clustering are well-established partitional and hierarchical methods respectively [32], while DBSCAN and GMM offer density-based and probabilistic alternatives suited to datasets with irregular cluster geometries [33]. Each algorithm was tested under four conditions—single-parameter (eGFR) with and without outliers, and two-parameter (eGFR and BUN) with and without outliers—yielding sixteen test configurations per sex. Algorithm performance was evaluated using the Silhouette Score, which measures the degree of separation between clusters. Unsupervised clustering has been demonstrated as an effective approach for identifying clinically meaningful patient subgroups from medical data without requiring labelled training sets [34]. All configurations were constrained to produce five clusters, corresponding to the five CKD stages.

### 2.7. Determination of Diagnostic Ammonia Thresholds

Following the parallel development of male and female clustering algorithms, the resulting stage-specific ammonia concentration ranges were derived independently for each sex and subsequently embedded as separate diagnostic algorithms within the hardware prototype. Following clustering, the ammonia value distributions within each cluster were analyzed to derive stage-specific concentration ranges. Four threshold variables were manually optimized iteratively to maximize the proportion of data points correctly assigned to each stage group. The intervals defined by these four variables were then adopted as the diagnostic ammonia ranges for each CKD stage and embedded into the hardware algorithm.

### 2.8. Hardware Implementation

The hardware prototype was designed through a systematic component selection process guided by the functional requirements of the diagnostic algorithm. The primary sensing element was selected based on its sensitivity to ammonia at the concentration ranges established by the machine learning-derived thresholds. The microcontroller was chosen for its analog input capability, sufficient processing capacity for the embedded rule-based algorithm, and compatibility with the I2C display interface. The complete bill of materials, comprising the ammonia gas sensor, Arduino Nano microcontroller (Shenzhen Olearn Technology Co., Ltd., Shenzhen, China), I2C OLED display, lithium-ion battery, USB Type-C charging module, and 5 V step-up converter, was sourced from commercially available components at a total prototype cost of under IDR 1,500,000 (approximately USD 100). For mass production, per-unit costs are expected to decrease substantially due to economies of scale.

**Figure 2:**
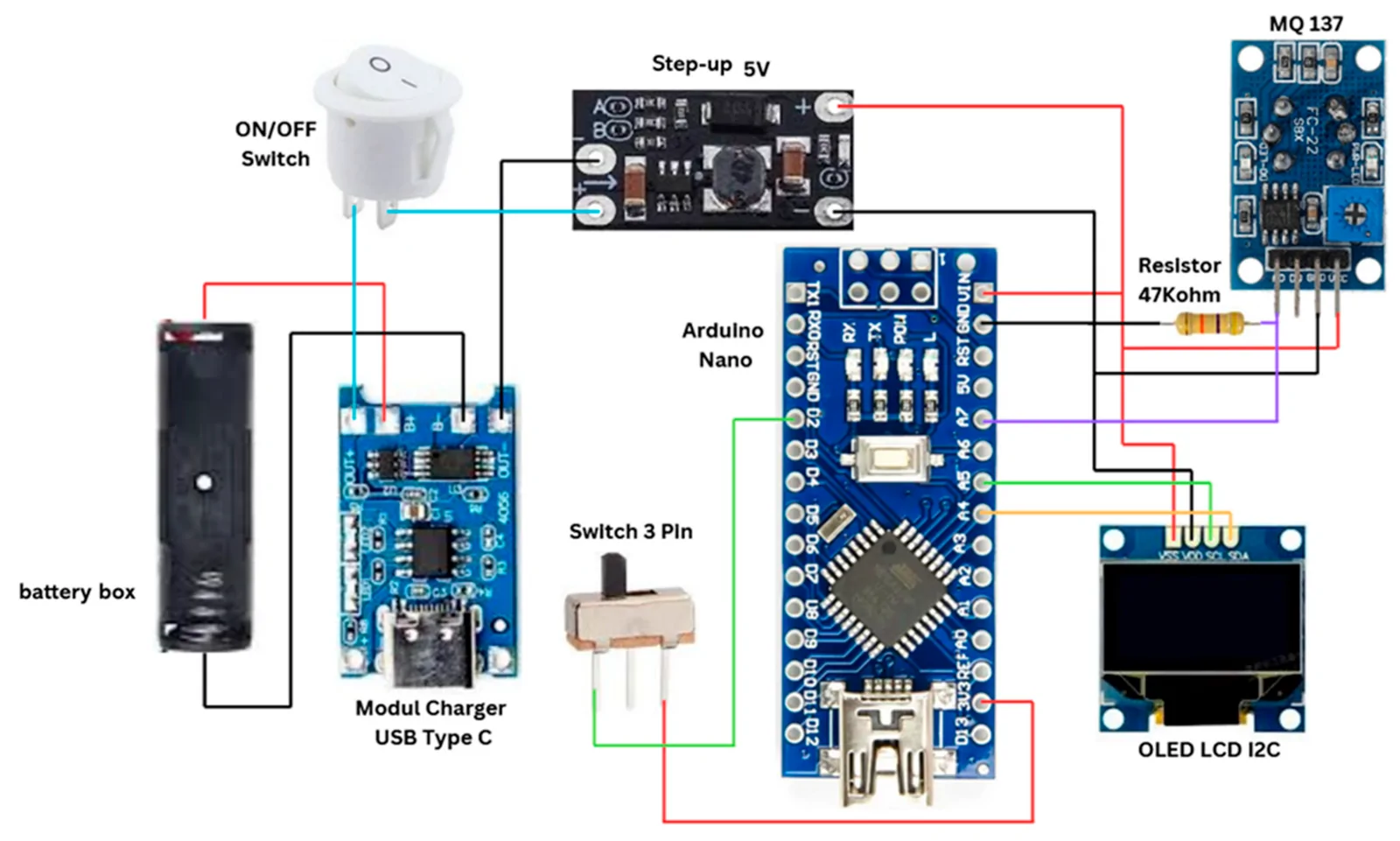
Complete Circuit Design.

The sensor calibration procedure follows established methodologies for metal oxide semiconductor gas sensors interfaced with Arduino-based platforms, with sensor characteristics derived from the manufacturer datasheet. Because the off-the-shelf MQ-137 sensor is nominally rated for higher ambient concentrations (≥5 ppm), a scaling factor of 10 was applied within the Arduino signal-processing code to adjust raw resistance outputs ($\frac{R_{s}}{R_{0}}$) to the sub-ppm and low-ppm range required for breath analysis. This empirical adjustment mapped digitized analog readings to the derived 250–3750 ppb threshold boundaries. The complete circuit design is shown in Figure 2.

### 2.9. Clinical Breath Sampling Protocol

To standardize sampling during hospital testing (n=29), patients adhered to a strict pre-test preparation protocol: 2-h pre-test fasting, restriction of high-protein foods/beverages, and a water-gargling cycle 15 min prior to breath collection to minimize oral salivary urea breakdown. Exhalation was standardized to a steady 5–8 s continuous breath into a sanitized, detachable mouthpiece. Ambient background readings were recorded prior to each trial to account for baseline environmental drift. Three consecutive trials were conducted per patient to determine modal classification.

## 3. Results

### 3.1. Clustering Performance

The Silhouette Scores obtained across all algorithms and condition combinations are summarized in Table 2 (single-parameter) and Table 3 (two-parameter). Across both male and female datasets, single-parameter clustering using eGFR alone consistently outperformed two-parameter clustering in terms of cluster separation. Among the four algorithms evaluated, K-Means, Agglomerative Hierarchical Clustering, and GMM produced identical Silhouette Scores under matched conditions, while DBSCAN showed greater sensitivity to outlier presence and parameter configuration.

Based on these results, K-Means Clustering with single-parameter input (eGFR) and retained outliers was selected for final threshold derivation, having achieved the highest Silhouette Scores of 0.582 (male) and 0.932 (female). The computational simplicity of K-Means relative to GMM and Agglomerative Clustering further supports its suitability for this application.

### 3.2. Stage-Specific Ammonia Thresholds

The stage-specific ammonia concentration ranges derived from the clustering results are presented in Table 4, separately for male and female algorithms. These threshold values were iteratively optimised to maximise within-stage coverage and are expressed in ppb. All ammonia input values to the embedded algorithm are divided by a factor of 10 prior to comparison against the stored thresholds, to align with the sensor’s measurable input range.

**Figure 3:**
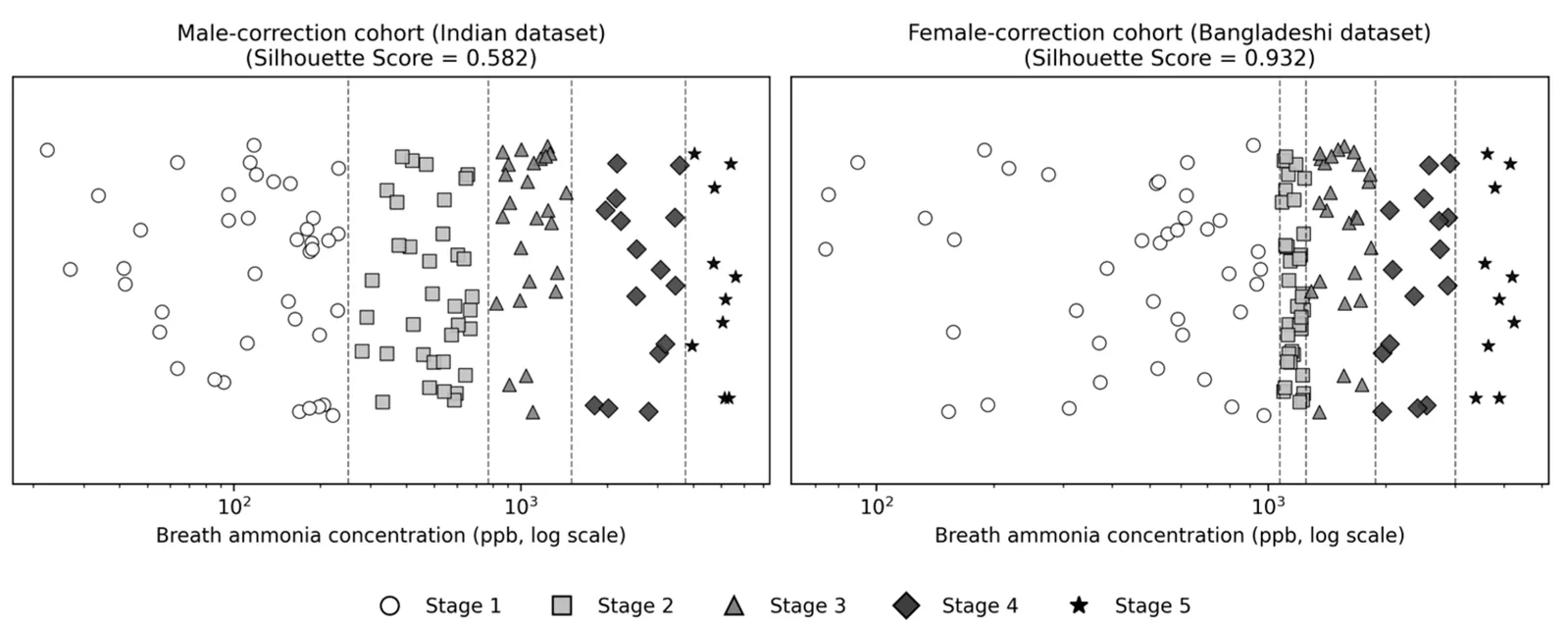
Per-patient breath ammonia concentration distribution (log scale) by CKD stage for the male-correction cohort (Indian dataset, **left**) and female-correction cohort (Bangladeshi dataset, **right**). Dashed vertical lines indicate Table 3 stage thresholds; markers are grayscale-coded by stage.

Figure 3 illustrates this per-patient ammonia distribution by stage for both cohorts, visually corroborating the threshold boundaries reported in Table 3. The female thresholds exhibit notably narrower inter-stage intervals compared to the male thresholds, particularly across Stages 1–3. This is consistent with the higher Silhouette Score observed for the female dataset (0.932 vs. 0.582 for male), indicating greater natural separation between CKD stages in the female feature space under single parameter eGFR clustering.

### 3.3. Algorithm Accuracy on Clinical Dataset

The stage classification accuracy of both algorithms was benchmarked against established clinical staging criteria derived from eGFR values. The male algorithm achieved a dataset-level accuracy of 82%, evaluated against the Indian hospital dataset, while the female algorithm achieved 92%, evaluated against the Bangladeshi hospital dataset. These figures represent the proportion of patients whose ammonia-derived stage classification matched their eGFR-derived clinical stage across their respective training datasets.

### 3.4. Hardware Prototype

The embedded diagnostic system consists of three functional blocks: power, sensing, and output. The power block comprises a lithium-ion battery, USB Type-C charging module, 5 V step-up converter, and on/off switch. The sensing block uses an ammonia gas sensor to capture exhaled breath concentration, with the analog output digitized via the microcontroller’s 12-bit ADC. The processing and output block consists of an Arduino Nano microcontroller and an I2C OLED display. A three-position switch allows the user to select between male and female diagnostic algorithms prior to measurement.

Arduino Nano processes the raw sensor signal through a calibration and normalization pipeline—converting analog readings to voltage, computing sensor resistance, and applying a logarithmic conversion to yield ammonia concentration in ppb. The calibrated value is then evaluated against the embedded stage-specific thresholds, and the resulting CKD stage classification is rendered on the OLED display in real time.

**Figure 4:**
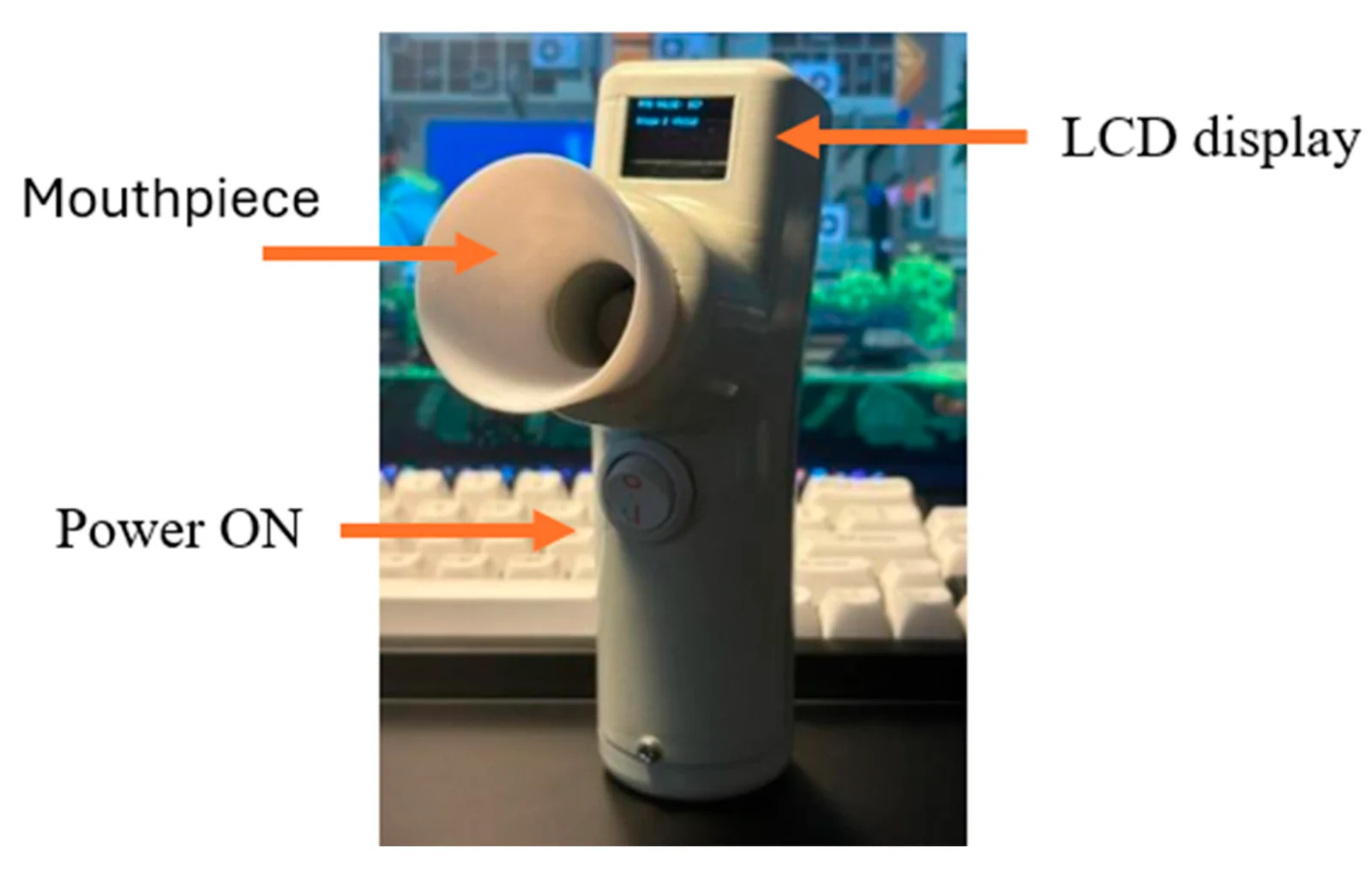
Hardware prototype used in this research.

The assembled prototype is shown in Figure 4. The device is compact and battery-powered, designed for handheld point-of-care testing (POCT). The OLED display presents the diagnosed CKD stage directly upon breath measurement, with sex-algorithm selection via the front-panel switch.

### 3.5. Hospital Validation

The prototype was evaluated in a hospital setting involving 29 patients in an Indonesian hospital, consisting of 22 male and 7 female patients, across different CKD stages. Each patient underwent three consecutive breath measurements to reduce individual variability, and the modal classification across the three readings was taken as the final output. Hospital testing yielded a modal classification accuracy of 90.5% (20/22; 95% CI: 71.1–98.3%) for the male-correction algorithm and 71.4% (5/7; 95% CI: 30.3–94.9%) for the female-correction algorithm. The wide confidence interval for female testing underscores the limitation of the small female sample size (n=7).

**Figure 5:**
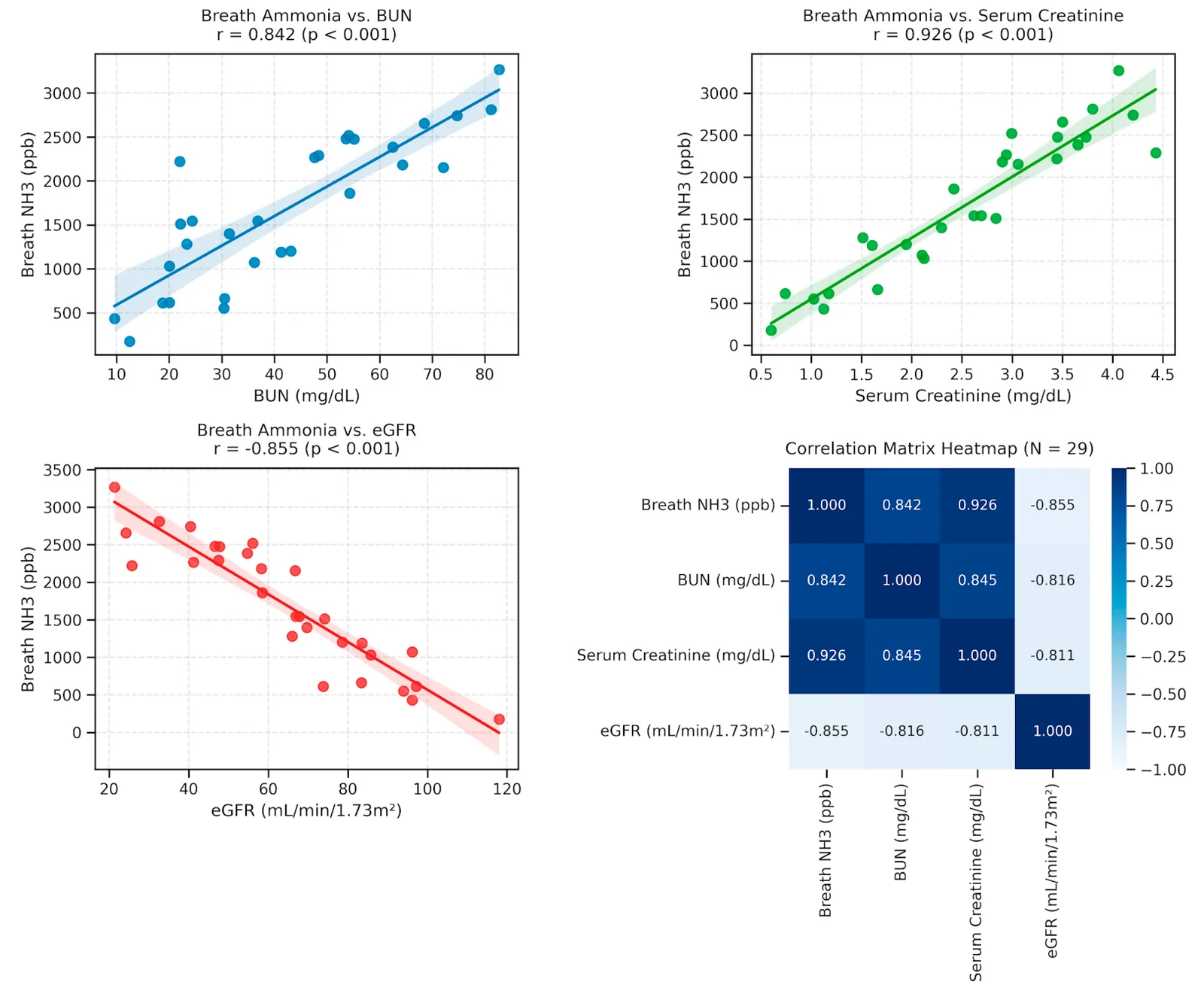
Graph showing the correlation between ammonia respiration and blood urea levels in kidney failure patients.

The correlation analysis from the N=29 hospital cohort demonstrates a strong, statistically significant association between measured breath ammonia (${\mathrm{NH}}_{3}$) and conventional renal function markers, aligning with and expanding upon findings from the prior literature. Figure 5 shows the exhaled ammonia exhibits strong positive correlations with blood urea nitrogen (r=0.842, p<0.001) and serum creatinine (r=0.926, p<0.001), alongside a strong inverse correlation with eGFR (r=−0.855, p<0.001). The BUN correlation exceeds the raw linear correlation reported by Chen et al. [35] (r=0.430), closely matching their log-transformed model (r=0.756) and the strong correlation observed by Chan et al. [36] (r=0.765). Furthermore, our serum creatinine and eGFR correlations demonstrate higher sensitivity than Chan et al. [36] (r=0.695 and r=−0.533, respectively), likely due to the higher proportion of advanced CKD inpatients in our cohort. These findings reinforce the physiological mechanism of salivary urea hydrolysis and support breath ammonia as a robust surrogate marker for monitoring renal impairment. Measured breath ammonia concentrations (ppb) demonstrate a statistically significant correlation with classical blood markers of renal function (BUN, serum creatinine, and eGFR), as shown in Table 5.

## 4. Discussion

The physiological mechanism linking breath ammonia to CKD severity relies on the accumulation of nitrogenous waste. In healthy individuals, the kidneys clear blood urea nitrogen (BUN); however, as kidney function deteriorates across Stages 1–5, impaired glomerular filtration causes systemic BUN elevation. Excess circulating urea diffuses into mucosal fluids and saliva, where it is hydrolyzed by bacterial salivary urease into gaseous ammonia (${\mathrm{NH}}_{3}$) and excreted via respiration. This biochemical cascade establishes the direct correlation between declining eGFR, rising BUN, and elevated breath ammonia concentrations observed across disease stages.

Regarding sex-specific variations, the differences in derived ammonia thresholds (Table 3) stem primarily from metabolic and physiological baseline discrepancies. Females generally exhibit lower average muscle mass, producing less endogenous creatinine than males for an equivalent level of renal impairment. The MDRD equation accounts for this by applying a 0.742 correction factor to female serum creatinine values. Consequently, when creatinine and BUN are converted to estimated breath ammonia, the corresponding eGFR boundaries shift, producing distinct, sex-adjusted staging thresholds. As shown in Table 3 and Figure 3, the female thresholds display narrower concentration intervals across Stages 1–3, reflecting the compressed eGFR-to-ammonia mapping under the female MDRD variant.

The results of this study demonstrate the feasibility of a low-cost, breath ammonia-based embedded system for non-invasive CKD staging, while also revealing important limitations that must be carefully considered in interpreting the findings.

The sex-adjusted clustering approach produced staging algorithms with meaningful discriminative performance at the dataset level 82% for the male-correction variant and 92% for the female-correction variant. The MDRD equation’s 0.742 female correction factor is clinically well-established and grounded in documented physiological differences in muscle mass and creatinine production between sexes [5]. However, because the two source cohorts also differ in geographic origin and population characteristics (India vs. Bangladesh), the distinct clustering behavior observed between them cannot be attributed to sex differences alone; it most likely reflects a combination of the MDRD correction term and inter-cohort population variation—a limitation discussed further below. These findings should therefore be read as evidence that sex-differentiated MDRD parameterization is clinically defensible [24], rather than as direct empirical confirmation that breath ammonia distributions differ by sex within a matched population. Preliminary prior work from this research group has explored male-only embedded breath ammonia classification [27,30]; the present system extends this by incorporating the female MDRD correction variant, though consistent with the dataset limitation noted above—this extension is best understood as a parameterization choice grounded in established clinical formulae rather than a population-validated sex-specific finding.

The clinical validation, while directionally encouraging, is constrained by its sample size. The hospital cohort of 22 male and 7 female patients is insufficient for robust statistical generalization, and the distribution of patients across CKD stages was not balanced, further limiting the interpretability of the reported accuracy figures. The female result—in particular 71.4% accuracy from 5 correctly classified out of 7 patients—carries wide confidence intervals and should not be read as a stable estimate of system performance. The discrepancy between this figure and the dataset-level algorithm accuracy of 92% is most plausibly attributable to sample size and imbalance rather than to a fundamental failure of the staging algorithm.

A notable methodological boundary of this study is the removal of non-CKD (healthy) entries prior to dataset clustering. Clinically, Stage 1 CKD is defined by an eGFR $\geq 90{\;\mathrm{mL}/\min/1.73\;m}^{2}$ accompanied by evidence of kidney damage (e.g., proteinuria or structural abnormalities). Because healthy individuals also exhibit an eGFR $\geq 90{\;\mathrm{mL}/\min/1.73\;m}^{2}$ and overlap significantly in baseline breath ammonia concentrations with Stage 1 patients, the current prototype cannot independently differentiate a healthy control from a Stage 1 CKD patient based on ammonia thresholds alone. Evaluating a healthy individual on the current device would categorize them into the Stage 1 threshold bin (≤250 ppb for males, ≤1070 ppb for females). Therefore, the prototype is intended for point-of-care staging and monitoring in patients already screened for renal risk. To adapt this system for broad community-wide screening, future iterations must integrate a healthy control cohort to establish a definitive ‘Healthy Normal’ baseline below Stage 1, potentially coupled with multi-biomarker sensing or urine test strip integration to verify kidney damage.

Beyond sample size, population heterogeneity represents an additional source of uncertainty. The training datasets were derived from South Asian cohorts in India and Bangladesh, whereas clinical validation was conducted in Indonesia. Dietary patterns are known to influence baseline breath ammonia levels through protein metabolism pathways [34], and meaningful differences exist between South Asian and Southeast Asian diets. This may introduce systematic offsets in absolute ammonia thresholds that are not captured by the current model. Furthermore, the clinical validation cohort comprised patients aged between 35 and 50 years, and demographic variables such as ethnicity were not recorded, limiting the ability to assess the influence of these factors on system performance. Locally calibrated threshold values and broader demographic data collection are therefore recommended in future validation studies.

Beyond clinical demographics, breath capture architecture plays a pivotal role in analytical accuracy. Recent breakthroughs in wearable breath analysis—such as smart masks for breath condensate harvesting [37,38], continuous biochemical monitoring platforms [39], and embedded synthetic biology sensors [38] highlight the critical role of standardized sample capture, fluidic transport, and controlled sensor exposure. Although our current prototype relies on passive manual exhalation through a sanitized 3D funnel, integrating standardized flow-control channels and active humidity/temperature management [40,41] will be essential in future hardware designs to further enhance diagnostic precision.

Situated within the broader literature, the present system occupies a deliberate position. Advanced breath ammonia platforms—including flexible semiconductor sensors achieving sub-100 ppb detection [19] and multi-analyte electrochemical facemask arrays [20]—offer substantially higher analytical precision but at a cost and complexity that renders them unsuitable for resource-limited settings. The trade-off made here is intentional: a device assembled from commercially available components at a fraction of the cost of laboratory instrumentation may enable screening in contexts where no alternative currently exists [22]. The system is not proposed as a replacement for blood-based clinical diagnostics, but as a non-invasive preliminary triage tool to identify individuals warranting further laboratory investigation—a role that, if validated at scale, could meaningfully complement existing CKD detection pathways in underserved communities.

It should also be clarified that the MQ-137’s detection limit is a property of its sensing material and cannot be overcome through analog signal amplification or downstream signal processing; below this limit, output variation reflects environmental noise rather than true ammonia response. The sensor was selected for this proof-of-concept prototype primarily for its low cost and commercial availability, consistent with the study’s goal of a sub-USD 100 point-of-care device intended for patients already identified as at renal risk, whose ammonia concentrations fall within the MQ-137’s usable response range (Stages 2 to 5). Improving the system’s low-concentration resolution for earlier-stage or healthy-control differentiation therefore requires either substituting the MQ-137 with a sensor of intrinsically lower detection limit (e.g., electrochemical or optical ammonia sensors), or introducing a breath sample pre-concentration step prior to sensing, both of which we identify as priorities for future hardware iterations.

## 5. Conclusions

This study presents a non-invasive, low-cost embedded system for CKD staging using exhaled breath ammonia measurement. While effective at stratifying disease severity among kidney disease patients, the system in its current implementation assumes prior clinical indication, as healthy individuals with normal eGFR share the Stage 1 ammonia threshold band. Future work will focus on: (1) recruiting healthy control cohorts to establish a distinct baseline threshold between healthy physiological ammonia levels and Stage 1 CKD; (2) replacement or supplementation of the MQ-137 sensor with higher-sensitivity sensing elements; and (3) expanding clinical trial validation across balanced populations. However, users and readers must note that threshold derivation was limited by the lack of individual sex labels in the training datasets, leaving inter-cohort differences confounded by regional, dietary, and genetic variations between the source populations. Broader generalization to other geographic populations including European and other non-Asian cohorts requires further validation and is identified as a priority for future work. Using K-Means clustering with single-parameter (eGFR) input, stage-specific ammonia threshold ranges were derived from independent clinical datasets and implemented within an Arduino Nano-based prototype equipped with an MQ-137 gas sensor. Dataset-level classification accuracy reached 82% for the male algorithm and 92% for the female algorithm. Hospital-based validation on 29 patients demonstrated a testing accuracy of 90.5% for male patients and 71.4% (5/7) for female patients.

The principal contribution of this work lies in extending breath ammonia-based CKD detection approaches to incorporate sex-adjusted. MDRD-based staging thresholds at the embedded hardware level and aspects not yet addressed in previously reported systems while maintaining the low-cost, portable form factor essential for point-of-care deployment in resource-limited settings. The results support the potential of this approach as an early-stage screening complement to conventional blood-based diagnostics, subject to the significant caveat that clinical validation remains limited by a small and imbalanced patient cohort.

Future work should prioritize: (1) replacement of the MQ-137 with a higher-sensitivity electrochemical or optical ammonia sensor, or incorporation of a breath sample pre-concentration stage to improve low-concentration resolution; (2) expansion of the clinical validation cohort to include balanced representation across all CKD stages and both sexes; (3) investigation of confounding factors including oral health, diet, and environmental ammonia exposure; and (4) exploration of multi-biomarker breath analysis to improve staging specificity beyond single-analyte ammonia measurement.

## Figures and Tables

**Table 1:** Comparison of Breath Ammonia and VOC Sensing Approaches for CKD Detection and Staging.

Sensing Technology	Primary Biomarker	Staging	Cost & System Complexity	Characteristics	References
SIFT-MS (*Selected Ion Flow Tube Mass Spectrometry*)	$\mathrm{Multi}\text{-}\mathrm{VOC}\;\mathrm{panel}\;(\mathrm{including}\;\mathrm{exhaled}\;NH_{3}$)	High analytical accuracy (AUC=0.902 for CKD differentiation)	Very high cost; bulky laboratory-grade equipment	Laboratory research; non-portable gold-standard clinical evaluation	Romani et al. [13]
UV-DOAS + Neural Network	$\mathrm{Exhaled}\;NH_{3}$ at ppb levels	Optical spectral reconstruction fitting for high precision	High cost; complex optical hardware setup	High-precision optical clinical diagnosis	Zhu et al. [17]
Semiconductive Polymer AI System	$\mathrm{Exhaled}\;NH_{3}$ biomarker	AI-assisted multimodal precision sensing	Moderate to high; requires specialized polymer fabrication	Multimodal laboratory/point-of-care hybrid testing	Weisbecker et al. [16]
$\mathrm{Ultrasensitive}\;\mathrm{Flexible}\;NH_{3}$ Sensor + SVM	$\mathrm{Exhaled}\;NH_{3}$ (LOD < 100 ppb)	Diagnostic accuracy: 0.93 (CKD identification) and 0.94 (staging)	Moderate cost; custom flexible sensor fabrication required	Early-stage CKD clinical screening and hemodialysis monitoring	Zhang et al. [19]
FFP2 Facemask Sensor Array	$\mathrm{Exhaled}\;NH_{3}$ + multi-VOC metabolites	Resistive sensor array integrated into disposable mask	Moderate cost; wearable disposable setup	Wearable, minimally invasive continuous monitoring	Capuano et al. [20]
Polypyrrole/Silver Nanoparticle Film Sensor	$\mathrm{Exhaled}\;NH_{3}$ gas	Conductive polymer nanocomposite film detection	Moderate cost; custom chemical film synthesis	Acute kidney injury/COVID-19 patient monitoring	Kamalabadi et al. [15]
Proposed Platform *(MQ-137 + Arduino Nano)*	$\mathrm{Exhaled}\;NH_{3}$ (Sex-adjusted MDRD thresholds)	Dataset Accuracy: 82% (Male), 92% (Female) Hospital Accuracy: 90.5% (Male), 71.4% (Female)	Ultra-low cost (<USD 100/IDR 1.5 M); fully portable, off-the-shelf components	Portable, non-invasive point-of-care preliminary screening in resource-constrained settings	This Study

**Table 2:** Silhouette Scores, single parameter (eGFR), all algorithms, male and female.

Algorithm Unsupervised Learning	Male				Female			
	with Outlier		Without Outlier		with Outlier		Without Outlier	
	Silhouette Score	Clusters	Silhouette Score	Clusters	Silhouette Score	Clusters	Silhouette Score	Clusters
K-Means	0.582	5	0.565	5	0.932	5	0.851	5
Agglomerative	0.582	5	0.565	5	0.932	5	0.851	5
DBSCAN	0.548	5	0.406	5	0.928	5	0.566	3
GMM	0.579	5	0.565	5	0.932	5	0.851	5

**Table 3:** Silhouette Scores, two-parameter (eGFR + BUN), all algorithms, male and female.

Algorithm Unsupervised Learning	Male				Female			
	with Outlier		Without Outlier		with Outlier		Without Outlier	
	Silhouette Score	Clusters	Silhouette Score	Clusters	Silhouette Score	Clusters	Silhouette Score	Clusters
K-Means	0.437	5	0.426	5	0.641	5	0.882	5
Agglomerative	0.43	5	0.402	5	0.614	5	0.882	5
DBSCAN	0.205	5	0.193	5	0.994	3	0.882	5
GMM	0.298	5	0.4	5	0.641	5	0.882	5

**Table 4:** Stage-specific breath ammonia concentration thresholds (ppb) for male and female diagnostic algorithms.

CKD Stage	Male (ppb)	Female (ppb)
Stage 1	X ≤ 250	X ≤ 1070
Stage 2	250 < X ≤ 770	1070 < X ≤ 1250
Stage 3	770 < X ≤ 1500	1250 < X ≤ 1880
Stage 4	1500 < X ≤ 3750	1880 < X ≤ 3000
Stage 5	X > 3750	X > 3000

**Table 5:** Measured breath ammonia concentrations (ppb) demonstrate a statistically significant correlation with classical blood markers of renal function (BUN, serum creatinine, and eGFR).

Renal Function Marker	Hospital Cohort (N = 29)	Chen et al. [7] (N = 125)	Chan et al. [8] (N = 244)
Blood Urea Nitrogen (BUN)	r = 0.842 (p < 0.001)	r=0.430 (p<0.001 ); log-transformed r=0.756	r = 0.765 (p < 0.001)
Serum Creatinine (sCr)	r = 0.926 (p < 0.001)	Not reported	r = 0.695 (p < 0.001)
eGFR	r = −0.855 (p < 0.001)	Not reported	r = −0.533 (p < 0.001)

## Data Availability

The datasets used in this study are publicly available. The male patient dataset is available at https://doi.org/10.24432/C5G020 and the female patient dataset at https://doi.org/10.24432/C5WP64.

## Abbreviations

The following abbreviations are used in this manuscript:

ADC	Analog-to-Digital Converter
AUC	Area Under the Curve
BUN	Blood Urea Nitrogen
CKD	Chronic Kidney Disease
DBSCAN	Density-Based Spatial Clustering of Applications with Noise
eGFR	Estimated Glomerular Filtration Rate
FFP2	Fitting Face Piece 2
GMM	Gaussian Mixture Model
MDRD	Modification of Diet in Renal Disease
ML	Machine Learning
NH_3_	Ammonia
OLED	Organic Light-Emitting Diode
POCT	Point-of-care testing
ppb	Parts per billion
ppm	Parts per million
ROC	Receiver Operating Characteristic
Rs/Ro	Sensor Resistance Ratio
VOC	Volatile Organic Compound
